# *IUCrJ* in 2025 – welcomes, farewells and special issues

**DOI:** 10.1107/S2052252525011194

**Published:** 2026-01-01

**Authors:** Andrew J. Allen

**Affiliations:** a Materials Measurement Science Division National Institute of Standards and Technology 100 Bureau Drive Gaithersburg MD 20899-8523 USA

**Keywords:** *IUCrJ*, quantum crystallography, cryoEM, metal–organic frameworks, MOFs, Nobel Prize in Chemistry

## Abstract

As we start a new year, it is timely to welcome two new Main Editors of *IUCrJ* and bid farewell to those who have stepped down, as well as highlight a couple of ongoing developments of interest to *IUCrJ* readers and authors.

As we start a new year, it is timely to welcome two new Main Editors of *IUCrJ* and bid farewell to those who have stepped down, as well as highlight a couple of ongoing developments of interest to *IUCrJ* readers and authors.

Two years ago, on the retirement of our founding Main Editor for the Chemistry & Crystal Engineering section, Professor Gautam Desiraju (Indian Institute of Science, Bangalore, India), Professor Susan Bourne (University of Cape Town, South Africa) valiantly agreed to step in to this position as his successor. We are most grateful for all the help and leadership she has provided to the journal. Increasingly active in her role on the IUCr Executive Committee, including very significant leadership contributions to the IUCr’s important ongoing outreach across all of Africa, Susan decided, earlier in 2025, that she needed to step down from her role as a Main Editor of *IUCrJ*. We are grateful to Phil Lightfoot, one of our retiring Co-editors, for taking on part of the Main Editor role while we conducted a search and then appointed a permanent successor, and we wish Phil well on retiring after his many years of service to the journal.

It is now a great pleasure to welcome Professor Leonard (Len) MacGillivray ▸ of Université de Sherbrooke, Quebec, Canada, as the Main Editor of *IUCrJ* for the Chemistry & Crystal Engineering section. Len has published more than 260 papers across a broad range of structural science, including metal–organic frameworks (MOFs); cocrystals; crystal engineering, growth and design; photodimerization; semiconductors; chemical crystallography; and self-assembly – indeed encompassing almost all areas relevant to the Chemistry & Crystal Engineering portfolio. Len has served previously as Chair of the Editorial Board of *CrystEngComm* and as a Topics Editor for *Crystal Growth and Design*, and he has also served as an active Co-editor of *IUCrJ* for several years, as well as being active with the IUCr Commission on Structural Chemistry. Len has already made major contributions to the editorial leadership of *IUCrJ*, and we look forward to working with him in the years to come.

In 2025, our founding Main Editor for the Biology & Medicine section, Professor Edward (Ted) Baker (University of Auckland, New Zealand) also stepped down from his role with *IUCrJ*. It is impossible to do justice here in describing Ted’s enormous contributions to the IUCr and its journals, with multiple roles spread over four decades. Ted served as the Section Editor of *Acta Cryst. D* for 11 years before moving to *IUCrJ* as founding Main Editor for the Biology & Medicine section in 2014. Over the years, Ted has worked with the IUCr journals, the Protein Data Bank (wwPDB) and other organizations to establish and promote high-integrity structural science publication and data curation in biology and medicine. As many will remember, he was also a very effective IUCr President from 1996 to 1999.

At *IUCrJ*, we now welcome our new Main Editor for the Biology & Medicine section: Professor Richard Garratt ▸ of the São Carlos Institute of Physics (IFSC), University of São Paulo, Brazil. Richard has published across molecular biology, biochemical sciences, medicine and structural science, with papers in several IUCr journals, including *IUCrJ*. For more than ten years, Richard has been involved as an Editor with the IUCr journals, especially with *Acta Cryst. D*. Richard has played a major role in establishing biological and medical structural science in Latin America, developing innovative and effective teaching tools for structural biology in the region. He set up the first protein crystallography laboratory in Brazil and worked with others to develop the first protein crystallography synchrotron radiation beamline in Latin America. Like Len, Richard is already significantly contributing to *IUCrJ*’s editorial leadership, and we look forward to working with him in the coming years.

In the *IUCrJ* 10th Anniversary Editorial (Allen, 2025 ▸), the increasing importance of special issues to all IUCr journals, including *IUCrJ*, was highlighted. A year later, it is a pleasure to celebrate the success of the virtual special issue on Quantum Crystallography, spread over several IUCr journals with some 50% of the papers in *IUCrJ*. In their Editorial, the Guest Editors (Paulina Dominiak, Angel Martín Pendás and Krzysztof Woźniak, 2025 ▸) introduce and provide links to all the papers across the journals included in the special issue (Dominiak *et al.*, 2025 ▸). Meanwhile, a new special issue, *CryoEM in the Fast Lane of Structural Biology*, is being assembled. We look forward to seeing many papers for this issue appear in *IUCrJ* over the next year.

Finally, we are delighted to announce that the 2025 Nobel Prize in Chemistry, awarded to Susumu Kitagawa, Richard Robson and Omar Yaghi for the discovery and development of metal–organic frameworks (MOFs), is also leading to a new special issue. The practice of structural science underlying MOF development is showed by each of the three Chemistry Nobel Laureates having published multiple times in IUCr journals (*e.g.* Abrahams *et al.*, 1996 ▸; Delgado-Friedrichs *et al.*, 2003 ▸; Kondo *et al.*, 1995 ▸). The new special issue will comprise papers associated with work presented at the recent MOF symposium held in Stockholm in connection with the Nobel Prize in Chemistry.

Whether or not papers are submitted as part of special issues, the scopes of all the IUCr journals have been revised over the past year to better clarify the distinctions between the journals. High-quality papers presenting detailed expositions of structural science and associated research results should be submitted to the relevant specialized IUCr journal. Papers containing high-impact results or having significant cross-cutting interest across different aspects of structural science may be submitted to *IUCrJ* and are subject to pre-review selection by the *IUCrJ* Main Editors. We hope this guidance helps authors to discern the appropriate IUCr journal for their papers, and we continue to look forward to publishing the highest quality structural science in all IUCr journals.

## Figures and Tables

**Figure 1:**
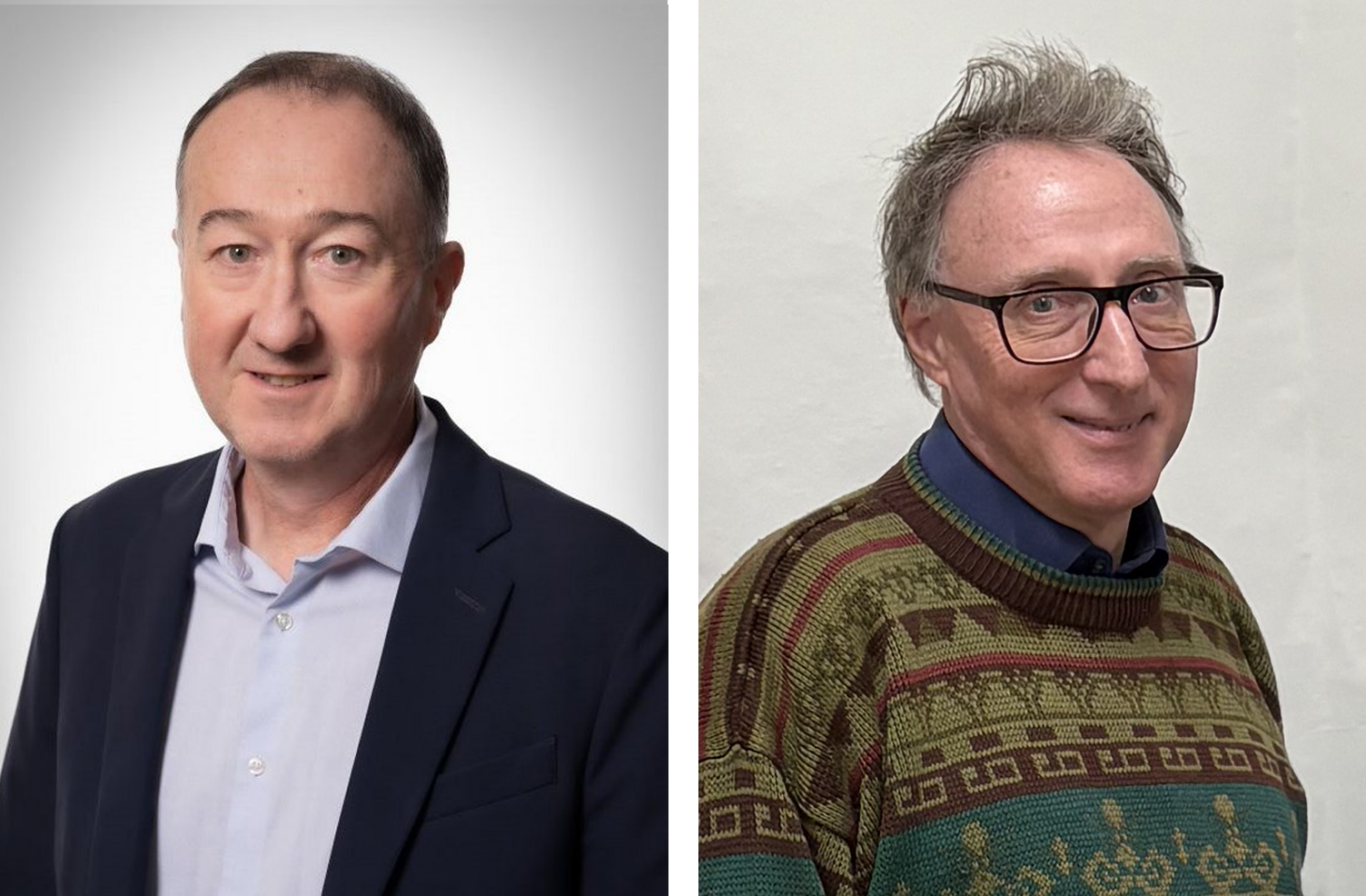
The new Main Editors of *IUCrJ*. Left: Professor Leonard (Len) MacGillivray, Université de Sherbrooke, Quebec, Canada, Main Editor for the Chemistry & Crystal Engineering section (image courtesy of Université de Sherbrooke). Right: Professor Richard Garratt, São Carlos Institute of Physics (IFSC), University of São Paulo, Brazil, Main Editor for the Biology & Medicine section (image courtesy of Professor S. S. Hasnain).
